# The effectiveness of an intervention designed based on health action process approach on diet and medication adherence among patients with type 2 diabetes: a randomized controlled trial

**DOI:** 10.1186/s13098-021-00773-x

**Published:** 2022-01-04

**Authors:** Soheila Ranjbaran, Davoud Shojaeizadeh, Tahereh Dehdari, Mehdi Yaseri, Elham Shakibazadeh

**Affiliations:** 1 Department of Health Education and Promotion, School of Public Health, Tehran University of Medical Sciences, Poursina Avenue, Tehran, Iran; 2 Health Promotion Research Center, Iran University of Medical Sciences, Tehran, Iran; 3 Department of Epidemiology and Biostatistics, School of Public Health, Tehran University of Medical Sciences, Tehran, Iran

**Keywords:** Adherence, HbA1c, Type 2 diabetes, Health action process approach, Iran

## Abstract

**Background:**

Diabetes is a major cause of worldwide morbidity and mortality. Diet and medication non-adherence are common among individuals with diabetes, making glycemic control difficult to attain. This study aimed to evaluate an intervention designed based on Health Action Process Approach (HAPA) to improve adherence to diet and medication among patients with type 2 diabetes in Tehran, Iran.

**Methods:**

The study was a randomized controlled trial. A total of 248 patients with type 2 diabetes who had low diet and medication adherence were randomly allocated into two intervention (n  = 124) and control (n  = 124) groups. Intervention group received educational intervention during three months. HAPA constructs, diet and medication adherence, and Hemoglobin A1c (HbA1c) levels were assessed at baseline, one month and six months after the intervention. Mixed Model Analysis was used to compare between and within group changes in the outcomes.

**Results:**

There was a statistically significant improvement in HbA1c levels after six months (7.77 ± 1.36% vs. 8.07 ± 1.52%, 95% CI, p  < 0.001). Diet and medication adherence, intention, task self-efficacy, coping self-efficacy, recovery self-efficacy, action and coping planning, barriers, benefits and perceived social support were significantly improved one month and six months after the intervention (p  < 0.001).

**Conclusion:**

Our intervention designed based on health action process approach led to improvements in diet and medication adherence, and HbA1c among the patients within one and six months.

*Trial registration:* IRCT, IRCT20151208025431N4. Registered 10 March 2018, https://fa.irct.ir

## Background

The number of people with diabetes has risen since 1980 to 422 million, according to the report of the World Health Organization (WHO) [1]. In Iran, prevalence of type 2 diabetes amongst adults was rapidly growing from 5.7% in 2010 to 14.3% in 2019 [2, 3]. Diabetes complications include heart attack, stroke, kidney failure, lower limb amputation [1].

Hemoglobin A1c (HbA1c) is a common health indicator of glycemic control. High level of HbA1c is related with the increased risk of diabetes related morbidity and mortality [4]. Management of type 2 diabetes requires active participation of patients in self-care behaviors, including prescribed diet and medication, which can lead to an improvement in HbA1c levels [5–9]. Risk of complications of diabetes can be reduced by proper adherence to diet and medications prescribed by doctors [10]. Interventions that aim to improve diet and medication adherence may result in better outcomes of glycemic control [11–14]. “Patient activation” has been conceptualized by Hibbard and colleagues as a new behavioral intervention strategy [15]. Active patients are people who have the knowledge, skills and confidence to manage their health [15]. Patient activation interventions have positive effects on glycemic control and healthy diet; and decrease HbA1c in patient with type 2 diabetes [16]. Theory-based interventions can help understand which specific techniques and approaches are effective to activate patients and why [17]. The health action process approach (HAPA) is an appropriate and effective model for active patient interventions in terms of including two phases of motivation and volition and self-emphasis [18].

## The health action process approach (HAPA)

HAPA describes the factors that influence adoption and maintenance of health behaviors [19]. According to HAPA, changing behavior involves two continuous phases: (1) a motivational phase including risk perceptions, outcome expectancies and task self-efficacy that lead to a behavioral intention; and (2) a volition phase that comes after a goal has been set within the motivation phase [20]. Volitional phase includes maintenance self-efficacy, recovery self-efficacy, action and coping planning that lead to the actual health behavior and is applied to bridge the gap between intentions and behaviors and perceived benefits and barriers (e.g., social support) [19, 21–25]. In this phase, peoples plan the details, try to act, invest effort, persist, possibly fail, and eventually recover [20]. Perceived benefits and barriers reflect pros and cons of performing the respective behavior in this study [25].

A number of interventions have used HAPA fruitfully to increase fruit and vegetable consumption [26–28]. However, few studies have examined the effect of HAPA-based interventions to improve adherence on diet and medication among patients with type 2 diabetes [27, 29–31]. Although the rate of adherence to diet and medication among patients with type 2 diabetes in Iran is low, a special program for adherence to diet and medication has not yet been included in the care program for patients in health centers. Then, such program can have a great effect in Iran, where the roles of health centers and health care providers in patient care are well established. Therefore, the present study aimed to evaluate an intervention designed based on HAPA to improve adherence to diet and medication among patients with type 2 diabetes.

## Methods

### Research design and participants

**Fig. 1:**
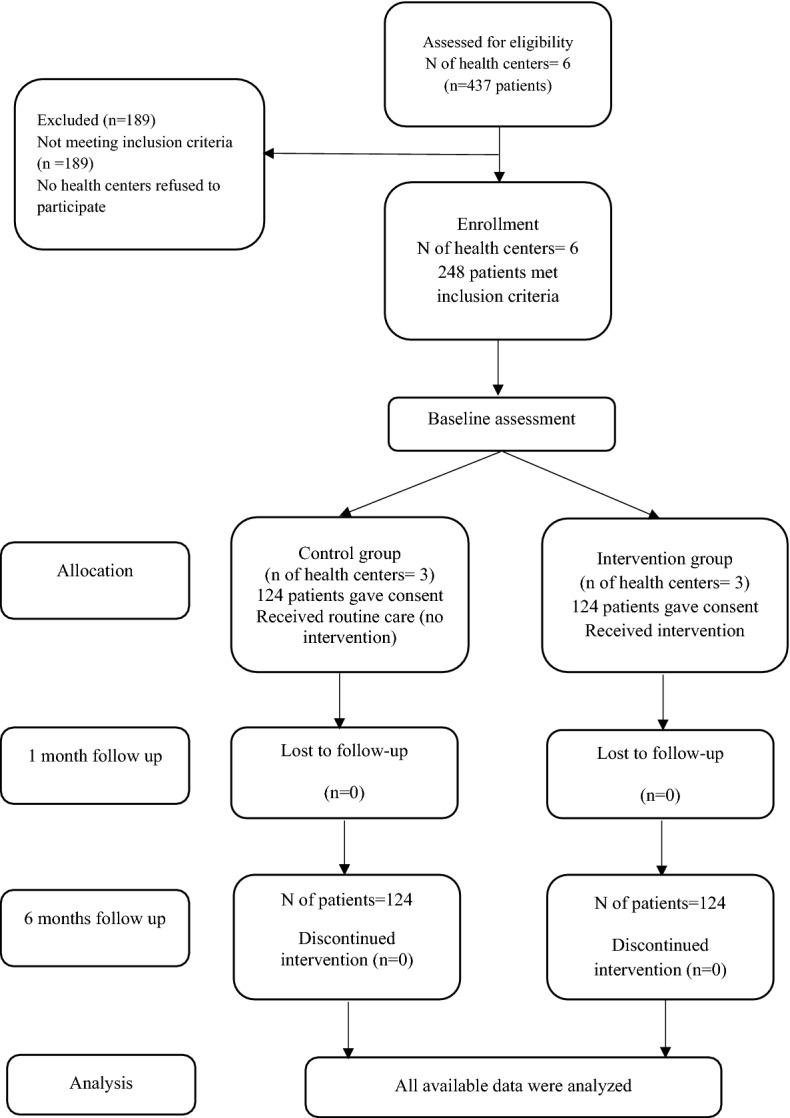
CONSORT trial flow chart

A cluster randomized controlled trial was conducted in South Tehran health centers during June–December 2018. In this study, 248 participants were recruited from six health centers. The health centers were randomly assigned into the intervention (three health centers) and control groups to have these 248 subjects we had to ask 437 patients registered to health centers,189 patients did not meet the inclusion criteria in the study. The participants were included in the study using simple random sampling Excel software. A total of 248 patients with Type 2 diabetes were randomly divided into the intervention (n  = 124) and control (n  = 124) groups. Study variables were measured at baseline, one and six months follow-up (Fig. 1).

The inclusion criteria were the onset of type 2 diabetes at least six months ago, aging less than 65 years, having non-adherence to diet (lower scores than six) and medication (lower scores than three), lack of other chronic diseases such as cancer, absence of any mental, visual, and learning disabilities (according to the clinical diagnosis by physician), no participation in similar classes offered by the healthcare centers. The exclusion criteria included having other types of diabetes including type 1 diabetes or gestational diabetes, lack of participation in at least half of our educational classes.

### Blinding

Blinding of data analyst and staffs laboratory were done.

### Sample size

Based on the results of a previous study [32], for a 90% power at 5% level of significance, to detect a (d  = 1) difference between two groups, the standard deviation of the intention score  = 1.37 when the drop-out rate was considered to be f  = 20%, and the design effect have assumed to be Deff  = 1.9, we needed to include 124 study participants in each study arm using the following formula. The final sample size was required as 248 participants.

$$ n = \frac{{\left( {Z_{1 - \alpha /2} + Z_{1 - \beta } } \right)^{2} \times \left( {S_{1}^{2} + S_{2}^{2} } \right)}}{{d^{2} }} \times Deff \times \frac{1}{1 - f} $$

### Measures

A HAPA self-structured questionnaire consisted of eight sections and 38 items and eight dimensions were developed. The dimensions consisted of: (a) intention to diet and medications adherence (4 items), measured using seven-interval Likert scales, ranging from 1 (strongly disagree) to 7 (strongly agree); (b) task self-efficacy of diabetes diet and medications adherence (14 items); (c) copping self-efficacy (13 items); (d) recovery self-efficacy (6 items); (e) action planning (5 items); (f) copping planning (13 items), rated on a 4-point scale ranging from 1 (not at all true) to 4 (exactly true); (g) barriers to adherence (18 items); (h) resources and benefits (8 items), scores for this item ranged from 1 (strongly disagree) to 7 (strongly agree), higher scores represented a high level of barriers. Content validity and reliability for the HAPA-based questionnaire has been measured in the previous studies [33, 34]. We transformed the scores of each item to 0–100 using the following formula: the new score of item  = 100 × (Score of item − Minimum possible score)/Range of possible scores. Then we calculated the section score as the average of item scores in that section for each sample.

The social support was measured using the “Chronic Illness Support Scale,” with diabetes patients’ family and friends’ subscales. Each item best indicated patients’ experience over the past three months. The family and friends’ subscales included eight items in a 5-point Likert scale (1 =  “not at all” to 5 =  “a great deal”). Internally consistent, test–retest reliability after two weeks for the subscales were α  = 0.75 and r  = 0.78, respectively [35].

We applied a reliable and valid nine-item scale to measure patients’ adherence to diet [36]. The total score ranged 0–9. The first seven items ranged from 0 (never), 0.33 (rarely), 0.66 (sometimes) to 1 (always).

The patients’ adherence to medication regimen was measured using the Persian version of MMAS-8-Item in Iran [36]. Each item in this questionnaire measures a specific medication-taking behavior. Responds categories were yes/no for each item and a 5-point Likert response for the last item [36]. The correlation coefficient was calculated by Negarandeh (*r*  = 0.8) and the Chronbach’s alphas was more than 0.7 [36]. All questionnaires were completed by one of the researchers through face-to-face interviews with the patients.

Glycated Hemoglobin (HbA1c) was determined using high-performance liquid chromatography and 7180 Clinical Analyzer (Hitachi, Japan).

### Intervention

The educational intervention was designed and implemented based on HAPA constructs (intention, task self-efficacy, coping self-efficacy, recovery self-efficacy, action planning, coping planning, barriers, resources and benefits and social support). The intervention group received intervention based on patient activation approach which focused on skill and confidence building during three months. The intervention was conducted through both group sessions and telephone calls. The intervention was held for 17 weeks, weekly 45–60-min group sessions. Participants received two booklets with the diet and medication adherence educational content and checklist of homework for planning. Monitoring planning were reviewed weekly and individualized feedback was given for planning. The first eight sessions provided the intervention goals, intention to diet and medication adherence, benefits of healthy behavior, increasing task self-efficacy, and coping self-efficacy strategies. The following eight sessions presented action and coping planning, ways to decrease barriers, recovery self-efficacy, preventing relapse and maintenance behavior of diet and medication adherence. Action and coping planning were presented in continued consecutive sessions. The action planning included determining when, where and how a behavior would be done, barriers ahead and coping strategies. Patients were asked to set plan for their diet and medication adherence, which their plans should include information about when, where, and how to adhere. Participants were also asked to plan how they would behave in the tempting situation. The following week, participants reported the success rate of their action and coping planning. In last session, we focused on perceived social support. Also, during the 3-month intervention, participants were contacted with telephone calls and feedbacks were provided about homework checklists and problems may have been encountered. The calls encouraged self-efficacy and planning, identification of probable barriers and copping strategies. Texts addressed medication and diet adherence, social supporting and other planning and self-efficacy behaviors. A combination of group sessions, in-person, telephone calls and delivering massages intervention were applied. At the end of the intervention, text messages were monthly delivered during 6-month follow-up to improve friends or family involvement about diet and medication adherence of their patients and patients were encouraged to continue their plan. Also, telephone calls were used to motivate and support implementation of individualized action planning in patients. Patients in control group received a usual program of diabetes management by health care providers in health centers (diabetes complications prevention and blood glucose monitoring).

The outcome measures: (a) primary outcomes: measures of diet and medication adherence and level of HbA1c, (b) secondary outcomes: measures of motivational phase (task self-efficacy, behavioral intention) and volitional phase (maintenance self-efficacy, recovery self-efficacy, action and coping planning, benefits and barriers of behavioral and social support).

### Statistical analysis

Demographic data and the outcome measures were reported using the means and standard deviations (SDs) for continuous variables; and frequency and percentages for categorical variables. Differences between two groups at baseline was evaluated by independent t-test and Chi-square tests. To compare the trend between two groups, we used interaction analysis of time and groups within hybrid linear mixed model. Using this analysis, we considered the correlation of the measurement in different centers and repeated measurements. All statistical analysis performed by SPSS (version 26.0). Statistical significance was considered for p value  < 0.05.

### Ethics statement

The Ethics Committee of Tehran University of Medical Sciences (code: IR.TUMS.SPH.REC.1396.4200) approved the study. Before enlistment in the study, the participants received a complete explanation of the plan and objectives of the study and those willing to participate provided written informed consent.

## Results

Most of the participants were female (61.3% of the intervention and 64.5% of control group). Regarding age of the participants, 52.4% of the intervention group and 80% of the control group were 56–65 years old. No significant differences were observed between both study groups for the demographic variables (Table 1). There were no differences between the intervention and control groups in outcome variables (diet adherence, medication adherence, HbA1c levels, HAPA constructs) at baseline, except for the barriers of diet adherence (t  = − 3.16, p  = 0.002). The intervention group reported a significantly higher level of barriers for diet adherence compared with the control group (79.2 ± 11.8 vs. 74.3 ± 12.7), respectively.

**Table 1:** Demographic characteristics of the study participants in both intervention and control groups at baselien (n  = 248)

Variable	Group	Intervention group n = 124 n (%)	Control group N = 124 n (%)	p value^a^
Age (years)	≤ 45	12 (9.7)	6 (4.8)	0.181
	46–55	47 (37.9)	41 (33.1)	
	56–65	65 (52.4)	77 (62.1)	
Gender	Female	74 (61.3)	80 (64.5)	0.599
	Male	48 (38.7)	44(35.5)	
Marital status	Married	114 (91.9)	106 (85.5)	0.214
	Single	2 (1.6)	2 (1.6)	
	Widowed	6 (4.8)	15 (12.1)	
	Divorced	2 (1.6)	1 (0.8)	
Level of income (Rls)	˂ 5,000,000	7 (5.6)	9 (7.3)	0.929
	50,00,000–1,00,00,000	24 (19.4)	24 (19.4)	
	10,00,000–20,00,000	90 (72.6)	89 (71.8)	
	> 20,00,000	3 (2.4)	2 (1.6)	
Employment status	Unemployment	1 (0.8)	1 (0.8)	0.801
	Retired	24 (19.4)	27 (21.8)	
	Clerk	3 (2.4)	6 (4.8)	
	Free job	21 (16.9)	19 (15.3)	
	Housewife	75 (60.5)	71 (57.3)	
Level of education	Illiterate	23 (18.5)	30 (24.2)	0.554
	Elementary	52 (41.9)	45 (36.3)	
	Middle school	20 (16.1)	22 (17.7)	
	High school	22 (17.7)	17 (13.7)	
	University degree	1 (0.8)	4 (3.2)	
	Other	6 (4.8)	6 (4.8)	
Disease duration (years)	≤ 5	50 (40.3)	50 (40.3)	0.239
	5.01–10	35 (28.2)	38 (30.6)	
	10.01–15	18 (14.5)	22 (17.7)	
	15.01–20	17 (13.7)	7 (5.6)	
	20.01 +	4 (3.2)	7 (5.6)	
Medications	Tablet	86 (69.4)	90 (72.6)	0.477
	Tablet and Insulin	28 (22.6)	21 (16.9)	
	Insulin	10 (8.1)	13 (10.5)	

^a^Pearson chi-squared test

The HbA1c levels decreased significantly in the intervention group 6 months after the intervention (− 0.51%; p  < 0.001) (Table 2).

**Table 2:** HbA1c levels in the intervention and control groups

	Group		Estimated change difference	95% CI		p value^a^
	Control	Intervention		Lower	Upper	
HbA1c (%)						
Baseline mean ± SD	8.34 ± 1.46	8.07 ± 1.52				0.15
6-month follow-up mean ± SD	8.56 ± 1.45	7.77 ± 1.36	0.52	0.44	0.59	p < 0.001

*HbA1c* glycated hemoglobin; *CI* confidence interval

^a^Interaction analysis of time and groups within a linear mixed model

Tables 3, 4 show three-level linear mixed models suggested that participants in the intervention group significantly increased their adherence to diet (61.8 ± 10.2, p < 0.001) and adherence to medication (80.5 ± 8.4, p < 0.001) as compared with the control group at one month after the intervention.

**Table 3:** Medication adherence and HAPA constructs among patients with type 2 diabetes before and after the intervention

Variable	Group	Baseline mean ± SD	1-month follow-up Mean ± SD	6-month follow-up mean ± SD	p value^a^
Medication adherence	Intervention	34.1 ± 20.6	80.5 ± 8.4	89.1 ± 6.8	p < 0.001
	Control	37.3 ± 18.5	42.2 ± 15.8	41.5 ± 13.5	
Intention	Intervention	72.7 ± 16	83.9 ± 11.5	95.4 ± 6.9	p < 0.001
	Control	76.8 ± 15.7	76.8 ± 15.5	76.3 ± 15.4	
Task self-efficacy	Intervention	55.3 ± 26.4	76.7 ± 17.2	86.9 ± 11.7	p < 0.001
	Control	56.8 ± 23.9	58.3 ± 23.1	59.1 ± 22.5	
Coping self-efficacy	Intervention	56.2 ± 28.9	79.1 ± 19.4	88.8 ± 12.1	p < 0.001
	Control	55.7 ± 24.8	58 ± 23.6	58.3 ± 23.1	
Recovery self-efficacy	Intervention	48.3 ± 30.2	65.6 ± 20.8	73.5 ± 19.1	p < 0.001
	Control	46.5 ± 30.8	46.4 ± 30.7	45.3 ± 30.6	
Action planning	Intervention	46.2 ± 28.7	70.8 ± 19.1	80 ± 16.7	p < 0.001
	Control	46.9 ± 29.4	46.8 ± 29.4	44.8 ± 29.2	
Coping planning	Intervention	53.9 ± 27.9	79.8 ± 16.3	87.5 ± 10.9	p < 0.001
	Control	53.9 ± 26	56.9 ± 23.8	56.3 ± 23.1	
Barriers to medication adherence	Intervention	70.5 ± 19.1	52.3 ± 17.6	41.4 ± 16.2	p < 0.001
	Control	67.8 ± 16.5	68.5 ± 16.1	68.6 ± 15.5	
Benefits of medication adherence	Intervention	60 ± 20.9	72.6 ± 16.4	79.8 ± 15.1	p < 0.001
	Control	60.8 ± 17.8	62.5 ± 16.5	60.1 ± 16.1	
Social support	Intervention	33.1 ± 16	46.8 ± 15.5	52.9 ± 14.2	p < 0.001
	Control	34.4 ± 15.4	35.4 ± 14.9	35.2 ± 14.9	

*SD* standard deviation

^a^Interaction analysis of time and groups within a linear mixed model

**Table 4:** Diet adherence and HAPA constructs among patients with type 2 diabetes before and after the intervention

Variable	Group	Baseline mean ± SD	1-month follow-up mean ± SD	6-month follow-up mean ± SD	p value^a^
Diet adherence	Intervention	16 ± 9.4	61.8 ± 10.2	75.7 ± 7.8	p < 0.001
	Control	16.3 ± 9.8	17.5 ± 9.4	17.8 ± 9.6	
Intention	Intervention	53.6 ± 10.4	71.7 ± 7.9	87.3 ± 6.8	p < 0.001
	Control	51.1 ± 6.2	53.4 ± 6.2	53.4 ± 6.4	
Task self-efficacy	Intervention	7.2 ± 12.3	40.1 ± 13.9	56.8 ± 12.9	p < 0.001
	Control	6.8 ± 8.7	7.3 ± 8.5	7.1 ± 8.3	
Coping self-efficacy	Intervention	6.5 ± 13	37.3 ± 12.8	55.8 ± 13.4	p < 0.001
	Control	5.3 ± 8.5	6 ± 8.4	6.1 ± 8.6	
Recovery self-efficacy	Intervention	3.4 ± 11.4	32.3 ± 12.7	51.1 ± 15.1	p < 0.001
	Control	2 ± 6.7	2 ± 6.7	1.9 ± 6.6	
Action planning	Intervention	6.8 ± 13.3	44 ± 14.2	62.4 ± 17.5	p < 0.001
	Control	6.5 ± 9.3	7.6 ± 8.9	8.4 ± 9.6	
Coping planning	Intervention	6.7 ± 11.5	47.4 ± 11.9	66.1 ± 12.5	p < 0.001
	Control	6.6 ± 7.7	9.3 ± 6.9	9.9 ± 7.2	
Barriers to diet adherence	Intervention	79.2 ± 11.8	63.3 ± 12.1	52.1 ± 12.3	p < 0.001
	Control	74.3 ± 12.7	77.5 ± 11.6	78.6 ± 10.9	
Benefits of diet adherence	Intervention	54.6 ± 18.4	70.9 ± 14.8	81.2 ± 12.9	p < 0.001
	Control	57 ± 16.1	57.7 ± 16	56 ± 15.6	

*SD* standard deviation

^a^Interaction analysis of time and groups within a linear mixed model

Patients in the intervention group showed significant improvements in HAPA constructs and diet and medication adherence one month after the intervention, including: higher levels of intention to diet (71.7 ± 7.9, p < 0.001) and medication (83.9 ± 11.5, p < 0.001), stronger task self-efficacy for diet (40.1 ± 13.9, p < 0.001) and medication (76.7 ± 17.2, p < 0.001), higher levels of coping self-efficacy to diet (37.3 ± 12.8, p < 0.001) and medication (79.1 ± 19.4, p  < 0.001), promoting recovery self-efficacy to diet (32.3 ± 12.7, p < 0.001) and medication (65.6 ± 20.8, p < 0.001) in time of setbacks, formed action planning for diet (44 ± 14.2, p < 0.001) and medication (70.8 ± 19, p < 0.001) adherence and coping planning (47.4 ± 11.9, p  < 0.001); (79.8 ± 16.3, p  < 0.001) respectively, lower levels of barriers diet (63.3 ± 12.1, p < 0.001) and medication (52.3 ± 17.6, p < 0.001) adherence behaviors, increasing benefits of diet (63.3 ± 12.1, p < 0.001) and medication (72.6 ± 16.4, p < 0.001) adherence and perceived social support for diet and medication adherence (46.8 ± 15.5, p < 0.001).

The results of the Mixed Model Analysis test revealed that the HAPA model led to the change in mean scores of intentions to diet (87.3 ± 6.8, p  < 0.001) and medication adherence (95.4 ± 6.9, p  < 0.001), task self-efficacy to diet (56.8 ± 12.9, p  < 0.001) and medication adherence (86.9 ± 11.7, p < 0.001), coping self-efficacy to diet (55.8 ± 13.4, p  < 0.001), and medication adherence (88.8 ± 12.1, p < 0.001), recovery self-efficacy to diet (51.1 ± 15.1, p  < 0.001) and medication adherence (73.5 ± 19.1, p < 0.001), action and coping planning to diet (62.4 ± 17.5, p < 0.001; 66.1 ± 12.5, p  < 0.001) and medication adherence (80 ± 16.7, p  < 0.001; 87.5 ± 10.9, p < 0.001), barriers to diet (52.1 ± 12.3, p < 0.001) and medication adherence (41.4 ± 16.2, p < 0.001) and benefits of diet (81.2 ± 12.9, p  < 0.001) and medication adherence (79.8 ± 15.1, p < 0.001) and social support (52.9 ± 14.2, p  < 0.001) in the intervention group compared with the control group six months after the intervention (Tables 3, 4).

## Discussion

Diabetes is a chronic disease that has been recognized as a main global public health challenge. Adherence to diet and medication is crucial in patient with type 2 diabetes and factors that lead to non-adherence should be put in intervention programs and healthcare policies. Therefore, the aim of this was to evaluate an intervention designed based on Health Action Process Approach (HAPA) to improve adherence to diet and medication among patients with type 2 diabetes.

In general, patient activation intervention based on HAPA model had a positive effect on diet and medication adherence behaviors among patients with type 2 diabetes. HAPA approach was useful in improving diet and medication adherence among patients with type 2 diabetes.

The adherence rates among patients with type 2 diabetes in Iran explored in health centers were low [33, 34]. It is important to encourage patients to monitor their own progress in order for long-term adherence to be successful [31]. The results of this study is in similarity to previous studies, in which activated patients practiced healthy behaviors including healthy diet, physical activity and adherence to medication and health-related outcomes [37–41]. A study conducted by Lin et al. in Iran, reported that a HAPA-based intervention increased adolescents’ intake of fruit and vegetables one month and six months following the intervention [26]. A systematic review conducted by Almutairi in 2020 showed that patient activation concept used in the theory-based interventions including HAPA, health belief model, social cognitive theory, and PRECEDE- PROCEED was effective in self- management behaviors (physical activity, healthy diet, food care and blood glucose self-monitoring) among patient with type 2 diabetes [16].

Our findings support results of the other studies that action planning and coping planning are effective approaches to improve health behaviors [18, 26]. Tailoring specific and personalized diet and medication plans may help patients achieve their improving adherence goals.

In the present study, we found participation of patients’ families and friends in the intervention sessions beneficiary. Social support can be an important component to promote diet and medication adherence behaviors among patients with chronic conditions. This finding is consistent with the results of previous studies [26, 42, 43]. Rotberg et al. has shown higher social support associated with lower level of HbA1c [44]. Another study conducted by Döbler et al. in 2018 about a theory-based telephone-delivered follow-up intervention including motivational interviewing and personalized action planning revealed improvements in level of physical activity and health status in patients with type 2 diabetes [18]. Miller et al., in another study showed no contact from intervention group during the 3-month-follow-up in prediabetes led to no significant different between groups in HAPA constructs except outcome expectancies [45]. It seems that follow-up calls and delivered messages in long-term interventions based on HAPA model can maintain the behavior in chronic conditions.

The results of present study indicated a reduction in the levels of HbA1c in the patients six months after the intervention. The results of a study conducted in Germany based on HAPA showed a decrease in the levels of HbA1c, 12 months after the intervention [18]. There is an association between level of HbA1c and complications of diabetes. Each 1% reduction in level of HbA1c decreases the risk of complications of type 2 diabetes such as in risk of 21% related to diabetes, 21% for deaths related to diabetes, 14% for myocardial infarction and 37% for microvascular complications [46]. The most improvements in level of HbA1c was seen in the interventions designed based on HAPA and empowerment models [16]. Applying a combination of group/in-person interventions with reminders such as telephone calls and delivered messages seems to be effective in decreasing the HbA1c in patients with type 2 diabetes.

## Strengths and limitations of the study

In this study, barriers and temptations of patients were considered that affect the practice of ones. Without these cognitive factors, intervention programs are ineffective. Action plans and coping plans were recorded weekly by collection of planning checklists instead of self-reporting planning. Another strength of this study, there was no drop-out patients in during intervention and follow ups. Therefore, this research was successful in patients participation.

Some limitations require to be addressed in this study. Results of these study may be biased by patients’ incorrect information. Diet and medication adherence measurements were not performed directly however diet and medication adherence questionnaires has been validated in measuring diet and medication adherence among diabetes in Iran.

## Suggestions for future research

The findings of this research can help health care providers to design effective programs to improve diet and medication adherence behaviors among patients with type 2 diabetes, and addressing problems nonadherence in these group of patients. Performing of such programs could be cost–benefit and inexpensive, because adherence to diet and medication can prevent and decrease complications of diabetes, so it is suggested to use this approach in future studies. This intervention targeted diet and medication adherence, future researches could target other combinations of health behaviors in patients with type 2 diabetes. Future research could apply longer follow-ups e.g., 12 or 24 months to determine the maintenance of diet and medication behaviors. The results in this study were for patients referring to the South Tehran health centers in Iran, so further research is needed to determine the generalizability to other area of Iran and Western countries improving diet and medication adherence programs.

## Conclusion

Our study showed effectiveness of an intervention designed based on HAPA approach plus family and friends support on diet and medication adherence in patients with type 2 diabetes. The combination of grouping, in-person approaches in the intervention and reminders led to reduction in HbA1c levels in patients six months after the intervention.

## Data Availability

The datasets used and/or analyzed during the current study are available from the corresponding author on reasonable request.

## Abbreviations

HAPA: Health action process approach
HbA1c: Hemoglobin A1c
95% CI: 95% confidence interval
WHO: World Health Organization
Persian version of MMAS-8-Item: Persian version of Morisky Medication Adherence Scale-8-Item
SD: Standard deviation
